# Atomoxetine modulates the relationship between perceptual abilities and response bias

**DOI:** 10.1007/s00213-019-05336-7

**Published:** 2019-08-05

**Authors:** Carole Guedj, Amélie Reynaud, Elisabetta Monfardini, Romeo Salemme, Alessandro Farnè, Martine Meunier, Fadila Hadj-Bouziane

**Affiliations:** 1 Present Address: INSERM, U1028, CNRS UMR5292, Lyon Neuroscience Research Center, ImpAct Team, 16 Avenue Doyen Lépine, 69500 Bron, France; 2 University UCBL Lyon 1, F-69000 Villeurbanne, France

**Keywords:** Monkey, Atomoxetine, Discrimination, Signal detection theory, Line of optimal response

## Abstract

Elucidation of how neuromodulators influence motivated behaviors is a major challenge of neuroscience research. It has been proposed that the locus-cœruleus-norepinephrine system promotes behavioral flexibility and provides resources required to face challenges in a wide range of cognitive processes. Both theoretical models and computational models suggest that the locus-cœruleus-norepinephrine system tunes neural gain in brain circuits to optimize behavior. However, to the best of our knowledge, empirical proof demonstrating the role of norepinephrine in performance optimization is scarce. Here, we modulated norepinephrine transmission in monkeys performing a Go/No-Go discrimination task using atomoxetine, a norepinephrine-reuptake inhibitor. We tested the optimization hypothesis by assessing perceptual sensitivity, response bias, and their functional relationship within the framework of the signal detection theory. We also manipulated the contingencies of the task (level of stimulus discriminability, target stimulus frequency, and decision outcome values) to modulate the relationship between sensitivity and response bias. We found that atomoxetine increased the subject’s perceptual sensitivity to discriminate target stimuli regardless of the task contingency. Atomoxetine also improved the functional relationship between sensitivity and response bias, leading to a closer fit with the optimal strategy in different contexts. In addition, atomoxetine tended to reduce reaction time variability. Taken together, these findings support a role of norepinephrine transmission in optimizing response strategy.

**Electronic supplementary material:**

The online version of this article (10.1007/s00213-019-05336-7) contains supplementary material, which is available to authorized users.

## Introduction

The locus cœruleus-norepinephrine (LC-NE) system is currently viewed as a key component of behavioral flexibility (Aston-Jones et al. 1999; Bouret and Sara 2004), energizing behavior during cognitive and/or physical effort (Robbins 1997; Raizada and Poldrack 2007; Bouret and Richmond 2009; Malecek and Poldrack 2013; Kalwani et al. 2014; Varazzani et al. 2015). For instance, the LC activity is positively modulated by the level of difficulty in a context involving a reward/effort trade-off (Varazzani et al. 2015), suggesting an involvement of the NE system in mobilizing resources to face challenges (Raizada and Poldrack 2007; Bouret and Richmond 2015). Accumulating evidence in behavioral studies manipulating NE transmission has demonstrated its impact on a variety of cognitive processes (Coull et al. 1995; Doucette et al. 2007; Robinson et al. 2008; Decamp et al. 2011; Baarendse et al. 2013). For example, atomoxetine, a NE-reuptake inhibitor that increases NE availability in the synaptic cleft, was found to improve executive control in healthy subjects performing a response inhibition task (Chamberlain et al. 2009). Both theoretical and computational models suggest that the LC-NE system tunes neural gain in brain areas to optimize cognitive processes (Servan-Schreiber et al. 1990; Aston-Jones and Cohen 2005; Eldar et al. 2013; Devilbiss 2018). We recently reported NE-dependent brain network reorganization with a reduction in functional connectivity within and between several networks at rest (Guedj et al. 2016). Similar tuning of brain activity could be dependent upon the LC-NE system to optimize cognitive processes (Harris and Thiele 2011; Rodenkirch et al. 2019). Optimizing refers to the refinement of response selection to maximize reward rate, depending on the context (Gold and Shadlen 2007; Bogacz 2007); (Summerfield and Tsetsos 2012). To date, direct empirical evidence demonstrating whether the NE-mediated effects reflect optimization of the performance, as suggested by the theoretical and computational models (Brown et al. 2005; Shea-Brown et al. 2008; Eckhoff et al. 2009), is scarce. Providing such evidence will help clarify the role of NE in cognitive functions.

Here, we tested the optimization hypothesis by assessing *perceptual sensitivity* and *response bias*—within the framework of signal detection theory (SDT) (Green and Swets 1966; Wickens 2001)—in monkeys performing a Go/No-Go discrimination task. Typically, *sensitivity* refers to the aptitude at discriminating a target stimulus in a noisy background, while *bias* reflects the extent to which one response (e.g., “target present”) is favored compared with another (e.g., “target absent”). In addition, we implemented Lynn and Barrett’s (2014) framework describing a functional relationship between sensitivity and bias that can be mathematically described as *the line of optimal response* (LOR) (Lynn and Barrett 2014; Lynn et al. 2015). This functional relationship considers the level of signal/noise interference (i.e., how hard or easy it is to discriminate the target stimulus) and is influenced by the outcome value of the task (i.e., the cost/benefit balance of each response type) and the target frequency (i.e., rate of signal occurrence). The LOR thus defines the amount of bias that maximizes *utility* (i.e., the net benefit earned over a series of responses) at any given sensitivity level for a specific environmental context. Here, we examined monkeys’ performance in a Go/No-Go discrimination task after injection of atomoxetine (ATX). In addition, we manipulated the task contingencies (i.e., level of signal/noise interference, target frequency, and outcome values) to modulate the relationship between sensitivity and bias measures. We also examined whether ATX modulated response time. Based on the optimization hypothesis, we predicted that enhancing NE transmission would modulate the functional relationship between sensitivity and response bias to bring the animals’ performance closer to the LOR.

## Methods

### Subjects

Four female rhesus monkeys (*Macaca mulatta*, 7 to 15 years of age, 6 to 10 kg) participated in this study. Animals had free access to water and were maintained on a food regulation schedule, individually tailored (70–90 kcal/kg/day) to maintain a stable level of performance for each monkey*.* Work complied with European Union Directive 2010/63/EU and was approved by French Animal Experimentation Ethics Committee #42 (CELYNE).

### Experimental setup

Monkeys were seated in a primate chair approximately 10 cm in front of a 19 in. high-resolution touchscreen. Stimuli were 13 Latin letters, white on a black background (size 10 × 10 cm), appearing one-by-one at the center of the touchscreen. The whole experiment, i.e., the presentation of the stimuli, delivery of reward, and behavioral data acquisition was controlled by Presentation® software (https://www.neurobs.com/).

### Behavioral task

**Fig. 1:**
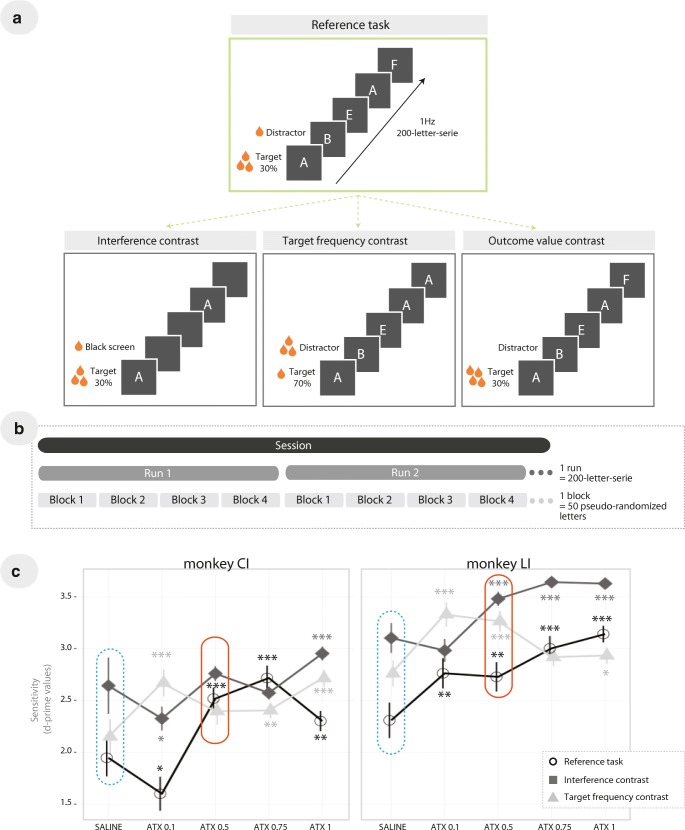
Behavioral task—**a** The reference task (i.e., 30% of target stimuli with distractors and larger reward for HITs (correct Go responses to targets) compared with correct rejection (correct No-Go withholding of response to distractors) and the other 3 task contrasts. Compared with the reference task, the other three variants of the task differed as follows: the interference contrast (black screens in place of letter distractors), the target frequency contrast (70% of target stimuli and smaller reward for HIT responses compared with correct rejection responses), and the outcome value contrast (increased reward for HIT responses and no reward for correct rejection responses). **b** Timeline of the task. A session was divided into runs that consisted of 200-letter series presented at a pace of 1 Hz. Each 200-letter series were pseudo-randomized in blocks of 50 letters, resulting in four blocks per run. **c** ATX dose-response curves (mg/kg) for sensitivity, for monkeys LI and CI. Results are plotted as mean ± SEM (one sample *t* test on Δ scores, i.e., percentage change from saline control condition (dotted blue boxes)—***p value < 0.0001; **p value < 0.001; *p value < 0.05). The smallest efficient dose was based on the performance in the reference task (orange boxes)

The task was a Go/No-Go continuous performance task designed to assess the ability to discriminate a target within a series of distractors (Decamp et al. 2011). The monkeys were trained to place their right hand on a starting point lever affixed to the chair to initiate the task and keep it running. The task consisted of a series of 200 letters (Fig. 1a)*.* For each series, one letter, the one appearing first, was the target, while the other 12 possible letters served as distractors. The monkey was required to touch the target (Go response) and to refrain from touching the distractors (i.e., to keep the hand on the starting point lever; No-Go response). Several 200-letter series were presented per testing session, each with a different target. Within each series, target and distractors were pseudo-randomized in order to enforce a target frequency of either 30% or 70% target letter presentations per block of 50 letters (Fig. 1b). A letter was presented for a maximum of 1 s. Correct responses led to a reward consisting of 1–15 drops of the animal’s favorite among a choice of slurries (applesauce, banana smoothie, vanilla milkshake, etc.). Incorrect responses were followed by a 3-s time out.

The Go/No-Go continuous performance task named here the “reference task” used as follows: (1) the presence of letter distractors, (2) a low target frequency (30%), and (3) unbalanced outcome value—50-ms valve opening time for each correct response to the rare target (HIT) and 35-ms valve opening time for each correct response to the frequent distractors (correct rejection (CR)). A longer valve opening time leads to a larger amount of reward compared with shorter valve opening time. To manipulate the task contingencies, hence the relationship between sensitivity and bias measures, we designed three “contrast” conditions of the task as follows (Fig. 1a): (1) an *interference contrast* replaced letter distractors by a black screen, (2) a *target frequency contrast* used a high target frequency (70%) with a reversal of the amount of reward attributed to correct responses compared with the reference task, and (3) an *outcome value contrast* where CRs were unrewarded and HITs were rewarded by a large amount that corresponded to a valve opening time of 150 ms.

Monkeys CE and CA were tested on one version of the task (the outcome value contrast). Monkeys LI and CI were tested on the other three versions of the task (the reference task, the interference contrast, and the target frequency contrast) presented in pseudo-random order within and between sessions. Each testing session was composed of a variable number of 200-letter runs (according to the monkey’s willingness to perform the task). As detailed in Table 1, monkeys completed 4 to 8 sessions. Each session lasted on average between 30 and 60 min. Each daily session ended when the monkey stopped responding during 10 consecutive min.

**Table 1:**
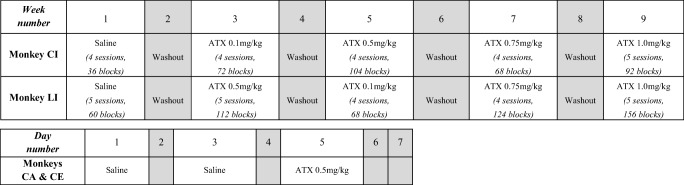
Drug administration schedule

Monkeys CI and LI were tested with either saline or ATX throughout a week, that included 4 to 8 sessions. The numbers in parenthesis indicates the numbers of blocks completed by each monkey for a given condition. Gray boxes represent washout periods, not included in the data analysis

Monkeys CE and CA were tested with either saline or ATX on different days across the week. Gray boxes represent days not included in the data analysis (washout periods). *[Monkey CA: 8 saline sessions, 132 blocks - 4 ATX sessions, 109 blocks; Monkey CE: 8 saline sessions, 201 blocks - 4 ATX sessions, 116 blocks]*

### Drug administration

After stable baseline performance was established, atomoxetine, a NE-reuptake inhibitor (ATX, Tocris Bioscience, Ellisville, MO) and saline (control) administration sessions began. The experimenter administered intramuscular injections of ATX or saline 30 min prior to testing (Gamo et al. 2010; Seu et al. 2009). For monkeys CI and LI, we tested four doses of ATX: 0.1, 0.5, 0.75, and 1.0 mg/kg. Each dose was administrated during 1 week, each separated by at least 7 days of washout. The smallest efficient dose in these two animals (0.5 mg/kg; see “Statistical Analysis” below and Fig. 1c) was then administered to the other two monkeys (CA and CE) with the following protocol per week: 1 day of ATX administration followed by 2-day washout and 1 day of saline control condition. Each monkey completed 4 to 5 ATX sessions with the 0.5 mg/kg dose and 4 to 8 saline sessions. The drug administration schedule for each animal is detailed in Table 1.

### Data analysis

We first computed, for each monkey and each task contrast condition, the HIT (% Go correct) and CR (% No-Go correct) rates per 50-trial blocks. We then computed a perceptual sensitivity index (*d*-prime—Eq. (1)) (Stanislaw and Todorov 1999), reflecting the subject’s ability to discriminate targets from distractors, and a response bias index (*c*—Eq. (2)) reflecting the subject’s tendency to respond by a “Go” or a “No-Go” (Stanislaw and Todorov 1999), two parameters taken from signal detection theory.

1 $$ {d}^{\prime }={\Phi}^{-1}\left(\mathrm{HIT}\ \mathrm{proportion}\right)-{\Phi}^{-1}\left(\mathrm{False}\ \mathrm{alarm}\ \mathrm{proportion}\right) $$

2 $$ c=-\frac{\Phi^{-1}\left(\mathrm{HIT}\ \mathrm{proportion}\right)+{\Phi}^{-1}\left(\mathrm{False}\ \mathrm{alarm}\ \mathrm{proportion}\right)}{2} $$

The Φ^−1^ function is the inverse of the normal cumulative distribution function.

A *c* value significantly superior to 0 reflected a “No-Go” bias whereas a *c* value significantly inferior to 0 reflected a “Go” bias.

Finally, we examined the median and standard deviation of the reaction times (RTs) for each 50-trial blocks. The standard deviation of RTs allowed assessing block-by-block variability in reaction times.

### Relationship between sensitivity and response bias: distance to the line of optimal response

We then investigated the relationship between sensitivity and bias. We estimated the *line of optimal response* (LOR) for each task contrast, i.e., the amount of bias that will maximize utility (maximize benefits and minimize costs) over *d-*prime values (i.e., *c*_optimal_) (Eq. (3)) (Lynn and Barrett 2014). Note that any given set of environmental target frequency and outcome values lead to a specific LOR. The optimal bias was defined as follows:

3 $$ {c}_{\mathrm{optimal}}=\frac{\log \left({\beta}_{\mathrm{optimal}}\right)}{d^{\prime }} $$

where *β*_optimal_ value (Eq. (4)) could be calculated from the target frequency and outcome values (Tanner Jr. and Swets 1954):

4 $$ {\beta}_{\mathrm{optimal}}=\frac{\left(1-\alpha \right)}{\alpha}\times \frac{\left(j-a\right)}{\left(h-m\right)} $$

where *α* is the target frequency and *j*, *a*, *h*, and *m* are the outcome values for correct rejections (CR), false alarms (FA), correct detections (HIT), and missed detections (MISS), respectively. Importantly, the outcome values’ array (*j*, *a*, *h*, *m*) was defined similarly across monkeys as objective values (Lynn et al. 2012; Lynn and Barrett 2014). Within this context, it is reasonable to assume that the benefits and costs associated with the different task contingencies could be ranked based on the objective outcomes from the lowest value to the highest value as 3-s wait (3-s time out) and no juice, 1-s wait and no juice, small amount of juice (valve opening time of 35 ms), middle amount of juice (valve opening time of 50 ms), large amount of juice (valve opening time of 150 ms). As such, the overall goal of the present experiment focused on the ability of ATX to change the perceiver’s distance to our estimate of the objective LOR rather than computing subjective utilities or individual, subjective LORs. For Eq. (4), we chose outcome values depending on the outcome values contrast conditions. We assigned the actual valve opening times in milliseconds to correct responses leading to a liquid reward, 0 for correct responses leading to no reward (1 s wait and no juice) and − 10 to incorrect responses leading to a penalty time and no reward (3-s time out). Thus, for the elements (*j*, *a*, *h*, *m*) of Eq. (4), we used (35, − 10, 50, − 10) for the reference task and interference contrast task, (50, − 10, 35, − 10) for the target frequency task where in addition to changing the frequency of target occurrence, we also rewarded CRs more than HITs responses, and (0, − 10, 150, − 10) for the outcome value contrast task, where reward was only delivered for correct “Go” responses. Then, we evaluated the Euclidean distance to the LOR for each pair of *d-*prime and *c* values to characterize how the monkeys adjusted their bias to their level of sensitivity (Lynn and Barrett 2014).

### Statistical analysis

#### Selection of the smallest efficient dose of ATX

The smallest efficient dose of ATX was determined in the reference task using the sensitivity index as an indicator of the subjects’ performance, as in previous literature (e.g., Coull et al. 1995). We computed *d-*prime values in the reference task for monkey CI and LI (Fig. 1c). Then, for each dose, *d-*prime values were normalized as the percent change from saline control condition:

$$ \mathrm{Individual}\ \Delta\ \mathrm{scores}=\frac{d{\prime}_{\left(\mathrm{ATX}\ \mathrm{dose}\ \mathrm{condition}\right)}-d{\prime}_{\left(\mathrm{mean}\ \mathrm{of}\ \mathrm{saline}\ \mathrm{condition}\right)}}{\mid d{\prime}_{\left(\mathrm{mean}\ \mathrm{of}\ \mathrm{saline}\ \mathrm{condition}\right)}\mid}\times 100 $$

One sample *t* tests were performed to determine whether these individual Δ scores significantly differed from 0.

#### Generalized linear mixed models

We examined the effect of the smallest efficient dose of ATX on the different variables computed above (i.e., HIT and CR responses, sensitivity, response bias, LOR, median and standard deviation of the reaction times) for each monkey, using generalized linear mixed models (“lmer” R-package). The predictor tested was the pharmacological condition, and for monkeys LI and CI, we also used the task contrast as an additional predictor. The term, *sessions*, was also included in the model as a random intercept. Post hoc comparisons were carried out using pairwise comparisons through the “emmeans” package for R (*p*-adjusted with the false discovery rate method (Lenth 2016). The behavioral data and the scripts are available as supplementary materials.

## Results

### Baseline performances

As shown in Table 2, in the control (saline) condition, the performance of the animals ranged from 57 to 100% correct for the HIT responses and from 69 to 92% correct for the CR responses. Two animals (LI and CI) were tested on three different versions of the task as follows: (1) the reference task with a low target frequency (30%) and distractors, (2) the interference contrast with a low target frequency (30%) and distractors, and (3) the target frequency contrast with a high target frequency (70%) and with distractors. In the saline condition, we found that, compared with the reference task, removing distractors or increasing the target frequency significantly enhanced performance, improving CR responses (*F*(2,29.31) = 17.35, *p* < 0.001 and *F*(2,56.93) = 6.53, *p* < 0.01, respectively for monkeys CI and LI). It also significantly improved the sensitivity index for monkeys LI (*F*(2,56.99) = 7.63, *p* < 0.01) and significantly modulated the response bias in both animals (*F*(2,28.89) = 19.45, *p* < 0.001 and *F*(2,56.98) = 13.64, *p* < 0.001, respectively for monkeys CI and LI). These results indicate that reducing interference and response inhibition improved performance and modulated the response strategy.

**Table 2:** Monkeys’ response types for all experimental conditions

Task contrast	Monkeys	% HIT					% CR				
		Saline	ATX 0.1 mg/kg	ATX 0.5 mg/kg	ATX 0.75 mg/kg	ATX 1 mg/kg	Saline	ATX 0.1 mg/kg	ATX 0.5 mg/kg	ATX 0.75 mg/kg	ATX 1 mg/kg
Reference	CI	87 ± 17	87 ± 17	95 ± 10**	99 ± 4***	97 ± 5***	81 ± 10	70 ± 15*	88 ± 8	88 ± 8	80 ± 10
	LI	98 ± 4	96 ± 9	95 ± 11	96 ± 8	98 ± 6	77 ± 19	89 ± 10***	89 ± 12***	93 ± 9***	95 ± 5***
Interference	CI	89 ± 16	96 ± 6*	97 ± 6**	99 ± 2***	100 ± 1***	92 ± 8	83 ± 9*	92 ± 5	85 ± 7	92 ± 6
	LI	100 ± 0	100 ± 1	99 ± 5	100 ± 1	100 ± 1	91 ± 11	91 ± 11	98 ± 4*	99 ± 2*	99 ± 1**
Target frequency	CI	95 ± 11	97 ± 6	98 ± 4	99 ± 1	99 ± 2	69 ± 10	83 ± 12**	73 ± 19	70 ± 14	81 ± 9**
	LI	99 ± 1	99 ± 2	99 ± 3	98 ± 3	98 ± 4	81 ± 13	94 ± 8***	93 ± 10***	87 ± 10*	88 ± 10*
Outcome value	CA	57 ± 36		67 ± 31***			80 ± 14		90 ± 6***		
	CE	88 ± 18		89 ± 19			91 ± 8		94 ± 6***		

Data are shown as means ± standard deviation computed across blocks. *HIT*, correct detection; *CR*, correct rejection (post hoc comparisons: statistical differences between saline and each ATX condition. ***p value < 0.001; **p value < 0.01; *p value < 0.05)

### Effect of ATX on response type, bias, and sensitivity

#### Smallest efficient dose of ATX

We then tested the effect of four ATX doses on the performance of monkeys CI and LI. The results on the animals’ response types (HIT and CR responses) for all the task contrasts and ATX doses are provided in Table 2. They show that both HIT and CR responses were differently modulated depending on the dose of ATX and the task contrast. The CR responses were significantly modulated by the pharmacological condition in both animals (*F*(4,356.47) = 4.12, *p* < 0.01 and *F*(4,488.81) = 16.68, *p* < 0.001, respectively for CI and LI) and the HIT responses were significantly modulated by the pharmacological condition in monkey CI (*F*(4,356.43) = 13.07, *p* < 0.001). To determine the smallest efficient dose of ATX, we computed a normalized sensitivity index (see “Statistical Analysis”). As shown in Fig. 1c, for monkey CI, the sensitivity to discriminate target from distractors was significantly impaired under ATX 0.1 mg/kg (*t*_(23)_ = − 2.1, *p* < 0.05), whereas it was improved for the three other doses compared with the saline (control) condition (all *p* values < 0.005; *t*_(31)_ = 5.4, *t*_(15)_ = 6.4, and *t*_(27)_ = 3.6, respectively for the doses 0.5, 0.75, and 1.0 mg/kg). For monkey LI, all ATX doses significantly improved the sensitivity compared with saline condition (all *p* values < 0.01; *t*_(23)_ = 3.1, *t*_(39)_ = 3.0, *t*_(43)_ = 5.8, and *t*_(55)_ = 10.3, respectively for the doses 0.1, 0.5, 0.75, and 1.0 mg/kg). Based on these results, we selected the ATX dose of 0.5 mg/kg as the smallest efficient dose for both animals. The other two monkeys, CA and CE, were only tested under 0.5 mg/kg ATX and the saline condition.

#### Sensitivity

**Fig. 2:**
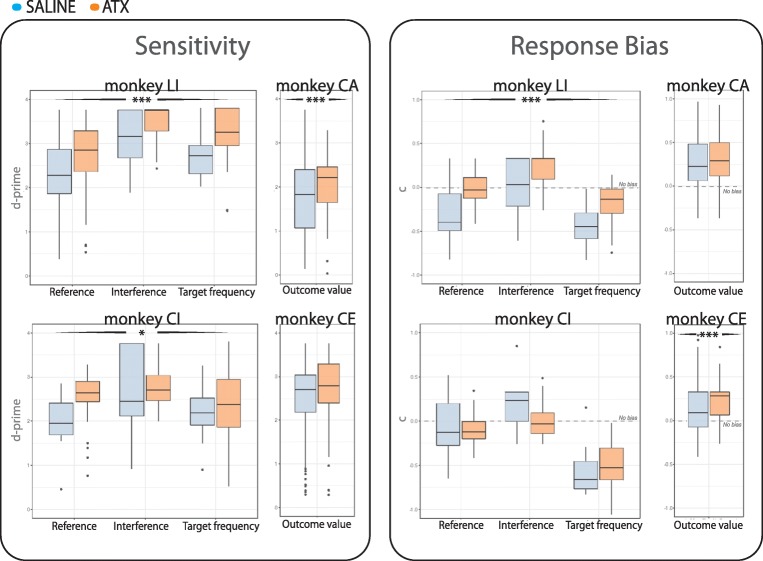
ATX effect on sensitivity and response bias—Sensitivity index (left panels) and response bias (right panels). For the boxplots illustrating response biases (right panels), the gray dashed line divides the *c* values according to “Go” (negative values) and “No-Go” (positive values) biases. Orange boxplots correspond to ATX 0.5 mg/kg conditions and blue boxplots correspond to saline (control) conditions. Black stars with arrow flankers indicate the main effect of statistical differences between saline and ATX 0.5 mg/kg conditions. (***p value < 0.001; **p value < 0.01; *p value < 0.05)

The boxplots in Fig. 2 (left panels) illustrate the sensitivity of each monkey and task contrast in both saline (blue) and ATX 0.5 mg/kg (orange) conditions. After ATX administration, the sensitivity to discriminate target stimuli was significantly improved in three out of four monkeys, regardless of the task contrast (monkey CI, *F*(1,134) = 6.03, *p* < 0.05; monkey LI, *F*(1,165.84) = 15.99, *p* < 0.001; and monkey CA, *F*(1,238.68) = 34.23, *p* < 0.001). In monkey CE, which reached ~ 90% correct on both HIT and CR responses in the saline condition, ATX did not improve sensitivity (*F*(1,280.55) = 2.14, *p* = 0.14).

#### Bias and response type

Figure 2 (right panels) illustrates the response bias of each monkey and task contrast in both saline (blue) and ATX 0.5 mg/kg (orange) conditions. ATX significantly affected the response bias in two of the four monkeys (i.e., monkeys LI and CE), regardless of the task demand. In both of these animals, boosting NE transmission tended to reduce or suppress the bias toward Go responses and/or increase the bias toward “No-Go” responses (*F*(1,165.49) = 44.51, *p* < 0.001 and *F*(1,305.92) = 12.92, *p* < 0.001, respectively for LI and CE). Accordingly, for these animals, monkeys LI and CE, only the CR responses were improved under ATX (post hoc comparisons are provided in Table 2 showing significant differences between saline and ATX 0.5 mg/kg for CR responses—*p* < 0.05—and no differences for HIT responses). For the other two monkeys, CI and CA for which ATX did not significantly change the response bias, we observed a significant improvement for the HIT responses (post hoc comparisons are provided in Table 2 showing significant differences between saline and ATX 0.5 mg/kg for HIT responses—*p* < 0.01). Monkey CA also improves its CR responses (*F*(1,152.14) = 56.63, *p* < 0.001).

Taken together, our results show that increasing NE availability improves sensitivity when the level of interference, response inhibition, or the outcome values are manipulated and could in addition influence the animals’ response bias by either reducing their bias toward Go responses and/or increasing their bias toward “No-Go” responses. We did not find any interaction between task and pharmacological conditions for response type, bias, or sensitivity.

### Relationship between sensitivity and response bias: Distance to the line of optimal response

**Fig. 3:**
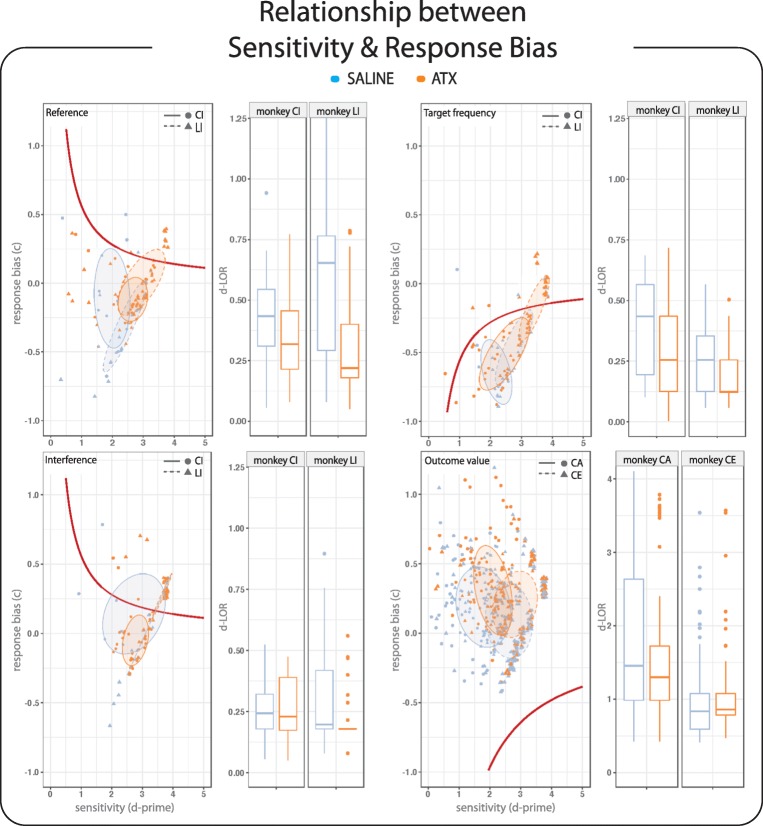
ATX effect on the distance to the line of optimal response (LOR)—Plots illustrating the relationship between sensitivity and response bias are depicted on the left. The red line represents the LOR for each task contrast, which depends on both the target frequency and the outcome values of the task. Each dot represents the average *d-*prime and *c* values for each block of a given task contrast and pharmacological condition (blue = saline and orange = ATX 0.5 mg/kg) for monkeys CI, CA (circles) and monkeys LI, CE (triangles). The ellipses surrounding the dots were drawn using a confidence level of 0.5. Adjacent boxplots on the right display the Euclidean distance to the LOR in each monkey and task contrast, in blue and orange, respectively for the saline (control) and ATX 0.5 mg/kg conditions

To examine the relationship between the sensitivity scores and the response bias and integrate them into the economic framework of decision-making, we modeled the LOR depending on the four task contrasts (see “Methods” for details). As shown in Fig. 3, the task contrast modifies the relationship between sensitivity and response bias resulting in different shapes of the LOR. Regardless of the task contrast, the LOR follows a general trend such that lower sensitivity scores are related to more pronounced bias. By definition, the LOR is tightly linked to the amount of expected utility (Lynn and Barrett 2014)—animals whose performance puts them closer to the LOR should obtain a more optimal balance of rewards and punishments.

We overlaid the animal performance (i.e., *d-*prime and *c* values) on the modeled LOR to estimate the Euclidean distance to this LOR. The boxplots in Fig. 3 display the Euclidean distances to a given LOR as a function of the task contrast for each monkey in both saline (blue) and ATX 0.5 mg/kg (orange) conditions. First, we found that, in the saline condition, the distance to the LOR varied across animals and was significantly impacted by task contingencies for monkey LI (*F*(2,56.94) = 7.88, *p* < 0.001). Second, after ATX administration, we found that the distance to the LOR was less variable and decreased in three out of four monkeys (monkey CI (*F*(1,134) = 5.53, *p* < 0.05), monkey LI (*F*(1,165.63) = 27.37, *p* < 0.001), and monkey CA (*F*(1,239) = 16.52, *p* < 0.001)). An interaction was found between task contrast and pharmacological condition in monkey LI (*F*(2,165.35) = 3.51, *p* < 0.05), revealing that ATX only affected the relationship between the sensitivity scores and the response bias on the reference task and the interference task contrast but not on the target frequency contrast. Overall, our results show that boosting NE transmission altered both sensitivity and response bias and their functional relationship bringing the animals’ performance closer to the line of optimal response.

## Effect of ATX on reaction times

**Fig. 4:**
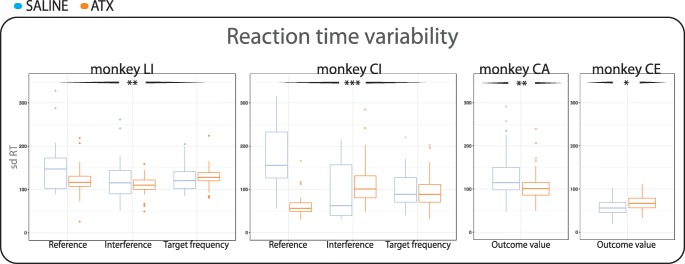
Standard deviation of reaction times—Box plots illustrate the standard deviation of reaction times in each monkey and task contrast under saline condition (blue) and ATX 0.5 mg/kg condition (orange). At the center of the plots are represented the median of the standard deviation of reaction times across blocks and dots represent outliers. Black stars with arrow flankers indicate the main effect of statistical differences between saline and ATX 0.5 mg/kg conditions. (***p value < 0.001; **p value < 0.01; *p value < 0.05)

RTs tended to increase and/or their variability tended to decrease after ATX (0.5 mg/kg) administration in all four monkeys. Specifically, RTs significantly increased in three out of four animals (monkey CI (*F*(1,131.38) = 29.50, *p* < 0.001), monkey LI (*F*(1,165.16) = 7.07, *p* < 0.01), and monkey CE (*F*(1,311.65) = 5.01, *p* < 0.05). RT variability decreased in three out of four monkeys, as shown by the significantly smaller standard deviation (Fig. 4) (monkey CI (*F*(1,132.88) = 15.47, *p* < 0.001), monkey LI (*F*(1,165.89) = 10.85, *p* < 0.01), and monkey CA (*F*(1,205.09) = 11.11, *p* < 0.01)). The only exceptions to the above finding about ATX effects were (1) in monkey CE, an increased RT variability (*F*(1,311.55) = 5.20, *p* = 0.02) and (2) in monkey CI, a RT increase restricted to the interference and target frequency task contrast (interaction (*F*(2,131.30) = 4.42, *p* = 0.01), and RT variability decrease restricted to the reference task (interaction (*F*(2,132.41) = 18.48, *p* < 0.001)).

## Discussion

We tested whether the modulatory effects following ATX injection in monkeys translate into an adjustment of the behavior toward the line of optimal response, reflected in the functional relationship between sensitivity index and response bias (Lynn and Barrett 2014). The animals’ performances were assessed in a Go/No-Go task under different task contingencies where we varied the level of signal/noise interference, the target frequency, and the outcome values. We found that boosting NE transmission tuned the functional relationship between sensitivity and response bias leading to a closer fit with the optimal strategy in the different task manipulations tested. Furthermore, under ATX, the subjects’ response time tended to increase and show less variability. Altogether, these findings support the hypothesis that enhancing NE availability optimizes response strategies.

## Boosting NE transmission fine tunes the functional relationship between sensitivity and response bias

In agreement with previous reports, we confirm that boosting NE transmission improved performance in a Go/No-Go task. This effect has been documented in humans and monkeys, on both correct detection (e.g. Coull et al. 1995; Decamp et al. 2011) and correct rejection (e.g. Usher et al. 1999). To tease apart some of the main components of the decision process that could be selectively affected by NE, we further manipulated the task contingencies (level of interference, target frequency, and outcome values) and analyzed perceptual sensitivity and response bias, in addition to simple accuracies (which confound the discriminability and bias elements of performance; Lynn and Barrett 2014). In the control condition (saline), increasing the level of interference and decreasing the target frequency altered the animals’ performance (monkeys LI and CI). After injection of ATX (a NE-reuptake inhibitor), the animals’ sensitivity index improved in all task contingencies. In other words, boosting NE transmission improves the sensitivity to discriminate a target stimulus whether or not the discrimination process involves interfering distractors, a rare or frequent event, or different outcome values. Future studies further manipulating the context might reveal NE-dependent contextual specificities. Two not mutually exclusive mechanisms might explain this pattern of results. The improvement in the different task variants might reflect a general arousal effect following ATX injection (Robbins 1997; Coull et al. 2004; Berridge et al. 2012) and/or the mobilization of energy or resources to face challenges (Raizada and Poldrack 2007; Malecek and Poldrack 2013; Kalwani et al. 2014; Bouret and Richmond 2015; Varazzani et al. 2015).

Does this improvement in terms of sensitivity scores following ATX injection reflect optimization of the animals’ response strategy? To address this question, we modeled the *line of optimal response* (LOR) for each task contrast, which describes the amount of bias needed depending on the subjects’ sensitivity. This relationship varies with the task contingencies (perceptual aspects of the decision and the outcome value associated with a given choice). The four animals did not perform the tasks with the same strategy in the control condition. Two animals (monkeys CE and LI) reached a high response rate in both HIT and CR responses while performances of the remaining two animals were lower (between 60 and 80% correct responses). As a result, ATX significantly modified the bias in the two animals that performed more poorly on CR compared with HIT responses (i.e., monkeys LI and CE). As suggested by Lynn and Barrett (2014), a given perceiver is able to adjust his bias to optimally accommodate his level of sensitivity. We found that ATX injection helps promote this adjustment, as previously inferred from the pupil size (Gee et al. 2014). In line with Lynn and Barrett’s (2014) the proposal, we found that this adjustment led to a closer fit of the performance with the LOR defined by the contingency of the task. The Euclidean distance between the performance and the LOR was reduced in the majority of the animals under ATX. One animal exhibited a significant interaction between task contingency and pharmacological condition for the distance to the LOR, suggesting that specificity based on the task at hand might emerge following a boost in NE transmission and future studies further manipulating the context might reveal NE-dependent specificities. Note that our experiment focused on manipulating the task contingency to change the perceiver’s distance to an estimate of the objective LOR using ranked values (Lynn et al. 2012; Lynn and Barrett 2014). While examining the relationship between individual NE receptor polymorphisms and the perceiver’s subjective distance to the LOR was beyond the scope of the current study, our results demonstrate that ATX reduced the perceiver’s distance to an estimate of the objective LOR. In Lynn and Barrett’s (2014) terminology, ATX might be changing the perceiver’s “subjective estimate” of the objective payoffs such that the perceiver values the payoffs differently in the two pharmacological conditions. It is equally possible that ATX affects the perceiver’s LOR by altering subjective values rather than its bias or sensitivity, per se. Optimization of behavior requires finding the best adjustment based on the evaluation of the different outcomes of given choices and the sensitivity and bias of the perceptual system and it is conceivable that the widespread projections of the LC-NE system, especially those directed toward the prefrontal cortex, influence or facilitate such computations as discussed in the next paragraph (Rich and Wallis 2016). Here, we suggest that the closer fit with the LOR following the NE challenge provides experimental support in favor of the role of NE in optimizing behavioral performance, in a constant environment. It would be interesting in future studies to assess the effect of ATX on individual’s subjective utility of gains and losses by systematically varying the levels of gains and losses and incorporating, for instance prospect theory, to translate objective into subjective gain and loss differences (Kahneman and Tversky 2012).

## How does enhanced NE availability adjust the performance in a perceptual discrimination task?

The optimization of the response strategy found in the ATX condition was accompanied in the majority of the cases by increased RTs and/or decreased RT variability. Increased RT measures could reflect a prolonged period during which information about the stimuli is accumulated before an option is selected (Bogacz et al. 2010). Trial-by-trial variability is considered a hallmark of how we select an option over multiple choices (Bellgrove et al. 2004; Johnson et al. 2007). A recent study by Murphy et al. (2014) found a correlation between variation in pupil diameter (considered as a proxy of the LC activity) and response time variability in perceptual decision-making in humans. In humans and rats, a series of experiments have demonstrated that NE agents (alpha-2-agonists or NE-reuptake inhibitors) affect response inhibition and response time variability (e.g. Bari and Robbins 2013). In the present study, we also altered NE transmission with a NE-reuptake inhibitor (ATX). In most of the task variants, the target appeared in 30% of the trials such that in most of the trials, the subjects were required to withhold their response. The decision to go or not to go had to be taken rapidly due to the frequency of stimulus presentation (≈ 1 Hz). On the one hand, the improvement in correct rejection (No-Go response) suggests that response inhibition was improved under ATX (e.g. Robinson et al. 2008; Chamberlain et al. 2009). On the other hand, the improvement in the HIT and the narrowing of the RT variability suggest an influence of ATX on attentional and/or decisional processes (Gee et al. 2014; Murphy et al. 2016; van den Brink et al. 2016). Within the framework of signal detection theory, our results highlight an improvement of the sensitivity to the target and the tendency of the animals to either shift their response bias toward a No-Go response or reduce their Go bias under ATX leading to a closer fit with the LOR. ATX acts by preventing the reuptake of NE in cortical and subcortical regions, leading to an increase of the post-synaptic effect of LC activation in each target area. The resulting increase in NE availability is also expected to act on LC inhibitory autoreceptors (Aghajanian et al. 1977) and reduce LC activity (Bari and Aston-Jones 2013). Our results suggest that together with a shift of the performance toward the LOR, ATX could also alter trial-by-trial variability in RTs. It is possible that this narrowing in RT distribution reflects an adjustment of the LC activity and neural gain to optimize performance (Servan-Schreiber et al. 1990). This adjustment could result in changes in functional connectivity at the whole-brain level, similar to those that we recently reported at rest (Guedj et al. 2016). In line with our recent proposal (Guedj et al. 2017), the effect reported here on the performance and response strategy optimization could be supported by NE-dependent local-to-global modulations of brain dynamics that depends on the context (de Gee et al. 2017).

## Conclusion

In the present study, implementing the utility-based approach to the signal detection theory (Lynn and Barrett 2014) that integrates both perceptual aspects of the decision and the outcome value associated with a given choice, we provide empirical evidence for a role of NE transmission in optimizing response strategy in a constant environment. Boosting NE transmission modified the functional relationship between sensitivity index and response bias leading to a closer fit with the optimal strategy in different contexts. It also tended to reduce the variability in reaction times. This neuromodulator, with widespread projections onto virtually the whole brain, facilitates behavioral adaptation in a variety of contexts. Here, we show that this facilitation results in fine tuning of the functional relationship between perceptual and decisional processes.

## Electronic supplementary material

ESM 1 (RTF 915 bytes)

ESM 2 (R 5 kb)

ESM 3 (RMD 16 kb)

ESM 4 (TXT 69 kb)

ESM 5 (TXT 95 kb)

ESM 6 (HTML 1483 kb)
