# Supplementary material for: Atomoxetine modulates the relationship between perceptual abilities and response bias
Source: Psychopharmacology (Berl). 2019 Aug 5;236(12):3641–53. doi: 10.1007/s00213-019-05336-7 (PMC6954008; doi:10.1007/s00213-019-05336-7)
Supplement: Supplementary file 1 — (RTF 915 bytes) [file 213_2019_5336_MOESM1_ESM.rtf]

——————————READ ME——————————- To compute the SDT metrics and the Distante to le Line of Optimal Response (LOR), you should run the ‘computeSDT.R’ code.- In this code, you could change the root path line 20, and the dataset to download line 23.- The LOR parameters are define line 108-180- The last line of this code (line 198) allows you to generate a Markdown html file with stats and plots (see the StatsPlots.Rmd)### R code by Carole Guedj - 05/14/2019
