# Supplementary material for: Atomoxetine modulates the relationship between perceptual abilities and response bias
Source: Psychopharmacology (Berl). 2019 Aug 5;236(12):3641–53. doi: 10.1007/s00213-019-05336-7 (PMC6954008; doi:10.1007/s00213-019-05336-7)
Supplement: Supplementary file 6 — (HTML 1483 kb) [file 213_2019_5336_MOESM6_ESM.html]

Stats and Plots


# Stats and Plots

### << RESPONSE TYPE >>

### —- HIT ——

## ci

```
## Type III Analysis of Variance Table with Satterthwaite's method
##                 Sum Sq  Mean Sq NumDF  DenDF F value   Pr(>F)   
## pharmaco      0.092369 0.092369     1 133.59 10.3784 0.001602 **
## task          0.047404 0.023702     2 133.07  2.6631 0.073448 . 
## pharmaco:task 0.019419 0.009710     2 133.20  1.0910 0.338878   
## ---
## Signif. codes:  0 '***' 0.001 '**' 0.01 '*' 0.05 '.' 0.1 ' ' 1
```

```
## $emmeans
## task = reference:
##  pharmaco    emmean         SE    df  lower.CL  upper.CL
##  SALINE1  0.8743758 0.02879690 66.55 0.8168898 0.9318619
##  ATX0,5   0.9541250 0.01837254 20.36 0.9158443 0.9924057
## 
## task = interference:
##  pharmaco    emmean         SE    df  lower.CL  upper.CL
##  SALINE1  0.8933063 0.02876137 67.64 0.8359083 0.9507043
##  ATX0,5   0.9699014 0.01759020 16.91 0.9327747 1.0070282
## 
## task = frequency:
##  pharmaco    emmean         SE    df  lower.CL  upper.CL
##  SALINE1  0.9545425 0.02879690 66.55 0.8970564 1.0120286
##  ATX0,5   0.9756479 0.01759400 16.89 0.9385091 1.0127867
## 
## Degrees-of-freedom method: kenward-roger 
## Confidence level used: 0.95 
## 
## $contrasts
## task = reference:
##  contrast            estimate         SE     df t.ratio p.value
##  SALINE1 - ATX0,5 -0.07974916 0.03237237 133.49  -2.463  0.0150
## 
## task = interference:
##  contrast            estimate         SE     df t.ratio p.value
##  SALINE1 - ATX0,5 -0.07659516 0.03181880 133.16  -2.407  0.0174
## 
## task = frequency:
##  contrast            estimate         SE     df t.ratio p.value
##  SALINE1 - ATX0,5 -0.02110540 0.03183598 133.31  -0.663  0.5085
```

## li

```
## Type III Analysis of Variance Table with Satterthwaite's method
##                  Sum Sq   Mean Sq NumDF  DenDF F value Pr(>F)
## pharmaco      0.0077804 0.0077804     1 165.40  2.1861 0.1412
## task          0.0165886 0.0082943     2 164.09  2.3305 0.1005
## pharmaco:task 0.0039722 0.0019861     2 163.50  0.5580 0.5734
```

```
## $emmeans
## task = reference:
##  pharmaco    emmean         SE    df  lower.CL  upper.CL
##  SALINE1  0.9800000 0.01520134 33.74 0.9490984 1.0109016
##  ATX0,5   0.9516250 0.01192083 13.83 0.9260271 0.9772229
## 
## task = interference:
##  pharmaco    emmean         SE    df  lower.CL  upper.CL
##  SALINE1  0.9927331 0.01580128 32.74 0.9605753 1.0248909
##  ATX0,5   0.9828087 0.01248159 15.80 0.9563220 1.0092955
## 
## task = frequency:
##  pharmaco    emmean         SE    df  lower.CL  upper.CL
##  SALINE1  0.9904505 0.01549446 33.28 0.9589368 1.0219643
##  ATX0,5   0.9856421 0.01248159 15.80 0.9591553 1.0121289
## 
## Degrees-of-freedom method: kenward-roger 
## Confidence level used: 0.95 
## 
## $contrasts
## task = reference:
##  contrast            estimate         SE     df t.ratio p.value
##  SALINE1 - ATX0,5 0.028375000 0.01633778 162.02   1.737  0.0843
## 
## task = interference:
##  contrast            estimate         SE     df t.ratio p.value
##  SALINE1 - ATX0,5 0.009924337 0.01701996 165.31   0.583  0.5606
## 
## task = frequency:
##  contrast            estimate         SE     df t.ratio p.value
##  SALINE1 - ATX0,5 0.004808426 0.01702526 165.28   0.282  0.7780
```

## ca

```
## Type III Analysis of Variance Table with Satterthwaite's method
##          Sum Sq Mean Sq NumDF DenDF F value    Pr(>F)    
## pharmaco 1.2859  1.2859     1   239  17.237 4.594e-05 ***
## ---
## Signif. codes:  0 '***' 0.001 '**' 0.01 '*' 0.05 '.' 0.1 ' ' 1
```

```
## $emmeans
##  pharmaco    emmean         SE   df  lower.CL  upper.CL
##  SALINE1  0.5164222 0.09274251 7.32 0.2990648 0.7337797
##  ATX0,5   0.7106792 0.09695368 8.60 0.4898019 0.9315564
## 
## Degrees-of-freedom method: kenward-roger 
## Confidence level used: 0.95 
## 
## $contrasts
##  contrast          estimate         SE  df t.ratio p.value
##  SALINE1 - ATX0,5 -0.194257 0.04719259 239  -4.116  0.0001
```

## ce

```
## Type III Analysis of Variance Table with Satterthwaite's method
##             Sum Sq   Mean Sq NumDF  DenDF F value Pr(>F)
## pharmaco 0.0050437 0.0050437     1 305.23  0.1643 0.6855
```

```
## $emmeans
##  pharmaco    emmean         SE    df  lower.CL  upper.CL
##  SALINE1  0.8709122 0.02424034  7.67 0.8145976 0.9272269
##  ATX0,5   0.8619775 0.02906727 12.83 0.7990964 0.9248585
## 
## Degrees-of-freedom method: kenward-roger 
## Confidence level used: 0.95 
## 
## $contrasts
##  contrast            estimate         SE     df t.ratio p.value
##  SALINE1 - ATX0,5 0.008934782 0.02234642 304.54     0.4  0.6896
```

### —- CR ——

## ci

```
## Type III Analysis of Variance Table with Satterthwaite's method
##                Sum Sq Mean Sq NumDF  DenDF F value    Pr(>F)    
## pharmaco      0.03351 0.03351     1 133.70  2.5669    0.1115    
## task          0.79651 0.39826     2 133.06 30.5023 1.247e-11 ***
## pharmaco:task 0.02440 0.01220     2 133.31  0.9342    0.3955    
## ---
## Signif. codes:  0 '***' 0.001 '**' 0.01 '*' 0.05 '.' 0.1 ' ' 1
```

```
## $emmeans
## task = reference:
##  pharmaco    emmean         SE    df  lower.CL  upper.CL
##  SALINE1  0.8043499 0.03463024 71.40 0.7353060 0.8733939
##  ATX0,5   0.8750000 0.02186825 22.78 0.8297373 0.9202627
## 
## task = interference:
##  pharmaco    emmean         SE    df  lower.CL  upper.CL
##  SALINE1  0.9170609 0.03460015 72.57 0.8480961 0.9860258
##  ATX0,5   0.9134000 0.02090607 18.77 0.8696062 0.9571938
## 
## task = frequency:
##  pharmaco    emmean         SE    df  lower.CL  upper.CL
##  SALINE1  0.6859333 0.03463024 71.40 0.6168893 0.7549772
##  ATX0,5   0.7257575 0.02090956 18.74 0.6819515 0.7695634
## 
## Degrees-of-freedom method: kenward-roger 
## Confidence level used: 0.95 
## 
## $contrasts
## task = reference:
##  contrast            estimate         SE     df t.ratio p.value
##  SALINE1 - ATX0,5 -0.07065007 0.03920546 133.61  -1.802  0.0738
## 
## task = interference:
##  contrast            estimate         SE     df t.ratio p.value
##  SALINE1 - ATX0,5  0.00366091 0.03854419 133.30   0.095  0.9245
## 
## task = frequency:
##  contrast            estimate         SE     df t.ratio p.value
##  SALINE1 - ATX0,5 -0.03982420 0.03855851 133.44  -1.033  0.3036
```

## li

```
## Type III Analysis of Variance Table with Satterthwaite's method
##                Sum Sq Mean Sq NumDF  DenDF F value    Pr(>F)    
## pharmaco      0.39521 0.39521     1 166.00 31.2804 9.024e-08 ***
## task          0.38904 0.19452     2 165.22 15.3962 7.389e-07 ***
## pharmaco:task 0.02531 0.01266     2 164.87  1.0018    0.3695    
## ---
## Signif. codes:  0 '***' 0.001 '**' 0.01 '*' 0.05 '.' 0.1 ' ' 1
```

```
## $emmeans
## task = reference:
##  pharmaco    emmean         SE    df  lower.CL  upper.CL
##  SALINE1  0.7670500 0.02672367 60.73 0.7136079 0.8204921
##  ATX0,5   0.8906500 0.01995736 23.14 0.8493792 0.9319208
## 
## task = interference:
##  pharmaco    emmean         SE    df  lower.CL  upper.CL
##  SALINE1  0.9175442 0.02791276 49.54 0.8614669 0.9736214
##  ATX0,5   0.9834500 0.02113912 25.83 0.9399844 1.0269156
## 
## task = frequency:
##  pharmaco    emmean         SE    df  lower.CL  upper.CL
##  SALINE1  0.8114497 0.02731069 54.91 0.7567157 0.8661837
##  ATX0,5   0.9276444 0.02113912 25.83 0.8841788 0.9711100
## 
## Degrees-of-freedom method: kenward-roger 
## Confidence level used: 0.95 
## 
## $contrasts
## task = reference:
##  contrast            estimate         SE     df t.ratio p.value
##  SALINE1 - ATX0,5 -0.12360000 0.03078273 162.05  -4.015  0.0001
## 
## task = interference:
##  contrast            estimate         SE     df t.ratio p.value
##  SALINE1 - ATX0,5 -0.06590585 0.03204415 165.98  -2.057  0.0413
## 
## task = frequency:
##  contrast            estimate         SE     df t.ratio p.value
##  SALINE1 - ATX0,5 -0.11619473 0.03206430 165.98  -3.624  0.0004
```

## ca

```
## Type III Analysis of Variance Table with Satterthwaite's method
##          Sum Sq Mean Sq NumDF  DenDF F value    Pr(>F)    
## pharmaco 0.6872  0.6872     1 152.14  56.631 4.286e-12 ***
## ---
## Signif. codes:  0 '***' 0.001 '**' 0.01 '*' 0.05 '.' 0.1 ' ' 1
```

```
## $emmeans
##  pharmaco    emmean         SE    df  lower.CL  upper.CL
##  SALINE1  0.7876523 0.01710501  8.77 0.7487996 0.8265051
##  ATX0,5   0.9210947 0.02017230 12.29 0.8772571 0.9649322
## 
## Degrees-of-freedom method: kenward-roger 
## Confidence level used: 0.95 
## 
## $contrasts
##  contrast           estimate         SE     df t.ratio p.value
##  SALINE1 - ATX0,5 -0.1334423 0.01842096 158.12  -7.244  <.0001
```

## ce

```
## Type III Analysis of Variance Table with Satterthwaite's method
##            Sum Sq  Mean Sq NumDF  DenDF F value    Pr(>F)    
## pharmaco 0.061771 0.061771     1 176.09   11.84 0.0007241 ***
## ---
## Signif. codes:  0 '***' 0.001 '**' 0.01 '*' 0.05 '.' 0.1 ' ' 1
```

```
## $emmeans
##  pharmaco    emmean          SE    df  lower.CL  upper.CL
##  SALINE1  0.9080655 0.005376727 12.35 0.8963877 0.9197433
##  ATX0,5   0.9370852 0.007565699 10.09 0.9202478 0.9539226
## 
## Degrees-of-freedom method: kenward-roger 
## Confidence level used: 0.95 
## 
## $contrasts
##  contrast           estimate          SE     df t.ratio p.value
##  SALINE1 - ATX0,5 -0.0290197 0.008853741 160.48  -3.278  0.0013
```

### << SDT PARAMETERS >>

### —- dprime ——

## ci

```
## Type III Analysis of Variance Table with Satterthwaite's method
##               Sum Sq Mean Sq NumDF DenDF F value   Pr(>F)   
## pharmaco      2.5961 2.59610     1   134  6.0311 0.015337 * 
## task          4.8216 2.41078     2   134  5.6005 0.004614 **
## pharmaco:task 0.9689 0.48443     2   134  1.1254 0.327575   
## ---
## Signif. codes:  0 '***' 0.001 '**' 0.01 '*' 0.05 '.' 0.1 ' ' 1
```

```
## $emmeans
## task = reference:
##  pharmaco   emmean        SE    df lower.CL upper.CL
##  SALINE1  1.946500 0.1925067 94.73 1.564312 2.328688
##  ATX0,5   2.518250 0.1159816 43.69 2.284457 2.752043
## 
## task = interference:
##  pharmaco   emmean        SE    df lower.CL upper.CL
##  SALINE1  2.642333 0.1931549 94.73 2.258858 3.025808
##  ATX0,5   2.759667 0.1101521 34.37 2.535900 2.983433
## 
## task = frequency:
##  pharmaco   emmean        SE    df lower.CL upper.CL
##  SALINE1  2.150000 0.1925067 94.73 1.767812 2.532188
##  ATX0,5   2.396000 0.1100764 34.37 2.172387 2.619613
## 
## Degrees-of-freedom method: kenward-roger 
## Confidence level used: 0.95 
## 
## $contrasts
## task = reference:
##  contrast           estimate        SE     df t.ratio p.value
##  SALINE1 - ATX0,5 -0.5717500 0.2247455 134.00  -2.544  0.0121
## 
## task = interference:
##  contrast           estimate        SE     df t.ratio p.value
##  SALINE1 - ATX0,5 -0.1173333 0.2215114 133.96  -0.530  0.5972
## 
## task = frequency:
##  contrast           estimate        SE     df t.ratio p.value
##  SALINE1 - ATX0,5 -0.2460000 0.2212102 133.96  -1.112  0.2681
```

## li

```
## Type III Analysis of Variance Table with Satterthwaite's method
##                Sum Sq Mean Sq NumDF  DenDF F value    Pr(>F)    
## pharmaco       7.1228  7.1228     1 165.84 15.9919 9.561e-05 ***
## task          15.9674  7.9837     2 165.25 17.9248 9.007e-08 ***
## pharmaco:task  0.1134  0.0567     2 165.05  0.1273    0.8805    
## ---
## Signif. codes:  0 '***' 0.001 '**' 0.01 '*' 0.05 '.' 0.1 ' ' 1
```

```
## $emmeans
## task = reference:
##  pharmaco   emmean        SE    df lower.CL upper.CL
##  SALINE1  2.308250 0.1558825 72.97 1.997575 2.618925
##  ATX0,5   2.727875 0.1147361 28.09 2.492883 2.962867
## 
## task = interference:
##  pharmaco   emmean        SE    df lower.CL upper.CL
##  SALINE1  3.111449 0.1629780 54.90 2.784820 3.438077
##  ATX0,5   3.483264 0.1219679 30.62 3.234383 3.732145
## 
## task = frequency:
##  pharmaco   emmean        SE    df lower.CL upper.CL
##  SALINE1  2.764013 0.1594095 63.33 2.445491 3.082534
##  ATX0,5   3.267458 0.1219679 30.62 3.018577 3.516339
## 
## Degrees-of-freedom method: kenward-roger 
## Confidence level used: 0.95 
## 
## $contrasts
## task = reference:
##  contrast           estimate        SE     df t.ratio p.value
##  SALINE1 - ATX0,5 -0.4196250 0.1827701 162.08  -2.296  0.0230
## 
## task = interference:
##  contrast           estimate        SE     df t.ratio p.value
##  SALINE1 - ATX0,5 -0.3718149 0.1901656 165.97  -1.955  0.0522
## 
## task = frequency:
##  contrast           estimate        SE     df t.ratio p.value
##  SALINE1 - ATX0,5 -0.5034451 0.1903362 165.96  -2.645  0.0090
```

## ca

```
## Type III Analysis of Variance Table with Satterthwaite's method
##          Sum Sq Mean Sq NumDF  DenDF F value    Pr(>F)    
## pharmaco 42.165  42.165     1 238.68  34.233 1.603e-08 ***
## ---
## Signif. codes:  0 '***' 0.001 '**' 0.01 '*' 0.05 '.' 0.1 ' ' 1
```

```
## $emmeans
##  pharmaco    emmean        SE   df    lower.CL upper.CL
##  SALINE1  0.7788145 0.3471264 7.38 -0.03347016 1.591099
##  ATX0,5   1.8881175 0.3655445 8.87  1.05940889 2.716826
## 
## Degrees-of-freedom method: kenward-roger 
## Confidence level used: 0.95 
## 
## $contrasts
##  contrast          estimate        SE     df t.ratio p.value
##  SALINE1 - ATX0,5 -1.109303 0.1915137 238.69  -5.792  <.0001
```

## ce

```
## Type III Analysis of Variance Table with Satterthwaite's method
##          Sum Sq Mean Sq NumDF  DenDF F value Pr(>F)
## pharmaco  1.553   1.553     1 280.55  2.1438 0.1443
```

```
## $emmeans
##  pharmaco   emmean         SE    df lower.CL upper.CL
##  SALINE1  2.435602 0.09499376  8.22 2.217541 2.653663
##  ATX0,5   2.590146 0.12138926 13.93 2.329664 2.850629
## 
## Degrees-of-freedom method: kenward-roger 
## Confidence level used: 0.95 
## 
## $contrasts
##  contrast           estimate        SE     df t.ratio p.value
##  SALINE1 - ATX0,5 -0.1545443 0.1076985 278.16  -1.435  0.1524
```

### —- bias ——

## ci

```
## Type III Analysis of Variance Table with Satterthwaite's method
##               Sum Sq Mean Sq NumDF  DenDF F value Pr(>F)    
## pharmaco      0.0748  0.0748     1 133.52  1.2966 0.2569    
## task          7.3677  3.6838     2 133.04 63.8255 <2e-16 ***
## pharmaco:task 0.3105  0.1552     2 133.13  2.6896 0.0716 .  
## ---
## Signif. codes:  0 '***' 0.001 '**' 0.01 '*' 0.05 '.' 0.1 ' ' 1
```

```
## $emmeans
## task = reference:
##  pharmaco      emmean         SE    df    lower.CL     upper.CL
##  SALINE1  -0.06710168 0.07363763 63.86 -0.21421590  0.080012531
##  ATX0,5   -0.09425000 0.04725685 19.17 -0.19309911  0.004599109
## 
## task = interference:
##  pharmaco      emmean         SE    df    lower.CL     upper.CL
##  SALINE1   0.18080314 0.07353452 64.89  0.03394002  0.327666256
##  ATX0,5   -0.01594254 0.04528245 15.99 -0.11194036  0.080055284
## 
## task = frequency:
##  pharmaco      emmean         SE    df    lower.CL     upper.CL
##  SALINE1  -0.57201835 0.07363763 63.86 -0.71913256 -0.424904136
##  ATX0,5   -0.50790660 0.04529314 15.97 -0.60393741 -0.411875789
## 
## Degrees-of-freedom method: kenward-roger 
## Confidence level used: 0.95 
## 
## $contrasts
## task = reference:
##  contrast            estimate         SE     df t.ratio p.value
##  SALINE1 - ATX0,5  0.02714832 0.08244168 133.43   0.329  0.7424
## 
## task = interference:
##  contrast            estimate         SE     df t.ratio p.value
##  SALINE1 - ATX0,5  0.19674568 0.08102287 133.08   2.428  0.0165
## 
## task = frequency:
##  contrast            estimate         SE     df t.ratio p.value
##  SALINE1 - ATX0,5 -0.06411175 0.08107307 133.25  -0.791  0.4305
```

## li

```
## Type III Analysis of Variance Table with Satterthwaite's method
##               Sum Sq Mean Sq NumDF  DenDF F value    Pr(>F)    
## pharmaco      2.6371 2.63711     1 165.49 44.5069 3.634e-10 ***
## task          5.3457 2.67287     2 164.43 45.1105 2.410e-16 ***
## pharmaco:task 0.0320 0.01598     2 163.96  0.2696     0.764    
## ---
## Signif. codes:  0 '***' 0.001 '**' 0.01 '*' 0.05 '.' 0.1 ' ' 1
```

```
## $emmeans
## task = reference:
##  pharmaco      emmean         SE    df    lower.CL    upper.CL
##  SALINE1  -0.29340000 0.06219798 33.08 -0.41993075 -0.16686925
##  ATX0,5    0.00917500 0.04886000 13.62 -0.09589751  0.11424751
## 
## task = interference:
##  pharmaco      emmean         SE    df    lower.CL    upper.CL
##  SALINE1   0.03052146 0.06463996 32.23 -0.10110959  0.16215251
##  ATX0,5    0.26365724 0.05113818 15.56  0.15499959  0.37231490
## 
## task = frequency:
##  pharmaco      emmean         SE    df    lower.CL    upper.CL
##  SALINE1  -0.43580771 0.06339085 32.69 -0.56482347 -0.30679195
##  ATX0,5   -0.17778720 0.05113818 15.56 -0.28644486 -0.06912954
## 
## Degrees-of-freedom method: kenward-roger 
## Confidence level used: 0.95 
## 
## $contrasts
## task = reference:
##  contrast           estimate         SE     df t.ratio p.value
##  SALINE1 - ATX0,5 -0.3025750 0.06666234 162.02  -4.539  <.0001
## 
## task = interference:
##  contrast           estimate         SE     df t.ratio p.value
##  SALINE1 - ATX0,5 -0.2331358 0.06944672 165.27  -3.357  0.0010
## 
## task = frequency:
##  contrast           estimate         SE     df t.ratio p.value
##  SALINE1 - ATX0,5 -0.2580205 0.06946803 165.25  -3.714  0.0003
```

## ca

```
## Type III Analysis of Variance Table with Satterthwaite's method
##              Sum Sq    Mean Sq NumDF DenDF F value Pr(>F)
## pharmaco 0.00013776 0.00013776     1 237.8   8e-04 0.9775
```

```
## $emmeans
##  pharmaco    emmean        SE   df  lower.CL  upper.CL
##  SALINE1  0.4842032 0.1210818 7.45 0.2013243 0.7670821
##  ATX0,5   0.4822039 0.1284542 9.15 0.1923454 0.7720623
## 
## Degrees-of-freedom method: kenward-roger 
## Confidence level used: 0.95 
## 
## $contrasts
##  contrast            estimate         SE     df t.ratio p.value
##  SALINE1 - ATX0,5 0.001999329 0.07178439 237.83   0.028  0.9778
```

## ce

```
## Type III Analysis of Variance Table with Satterthwaite's method
##          Sum Sq Mean Sq NumDF  DenDF F value    Pr(>F)    
## pharmaco 1.0728  1.0728     1 305.92  12.916 0.0003796 ***
## ---
## Signif. codes:  0 '***' 0.001 '**' 0.01 '*' 0.05 '.' 0.1 ' ' 1
```

```
## $emmeans
##  pharmaco    emmean         SE    df   lower.CL  upper.CL
##  SALINE1  0.1614024 0.04054066  7.65 0.06715632 0.2556484
##  ATX0,5   0.2918228 0.04839455 12.71 0.18703071 0.3966148
## 
## Degrees-of-freedom method: kenward-roger 
## Confidence level used: 0.95 
## 
## $contrasts
##  contrast           estimate         SE     df t.ratio p.value
##  SALINE1 - ATX0,5 -0.1304204 0.03677399 305.71  -3.547  0.0005
```

### —- dlor ——

## ci

```
## Type III Analysis of Variance Table with Satterthwaite's method
##                 Sum Sq  Mean Sq NumDF DenDF F value  Pr(>F)  
## pharmaco      0.157705 0.157705     1   134  5.5293 0.02016 *
## task          0.265563 0.132781     2   134  4.6555 0.01110 *
## pharmaco:task 0.064121 0.032061     2   134  1.1241 0.32800  
## ---
## Signif. codes:  0 '***' 0.001 '**' 0.01 '*' 0.05 '.' 0.1 ' ' 1
```

```
## $emmeans
## task = reference:
##  pharmaco    emmean         SE    df  lower.CL  upper.CL
##  SALINE1  0.4409167 0.04955284 94.73 0.3425382 0.5392951
##  ATX0,5   0.3347813 0.02985465 43.69 0.2746009 0.3949616
## 
## task = interference:
##  pharmaco    emmean         SE    df  lower.CL  upper.CL
##  SALINE1  0.2701667 0.04971970 94.73 0.1714569 0.3688764
##  ATX0,5   0.2621389 0.02835409 34.37 0.2045394 0.3197383
## 
## task = frequency:
##  pharmaco    emmean         SE    df  lower.CL  upper.CL
##  SALINE1  0.3955833 0.04955284 94.73 0.2972049 0.4939618
##  ATX0,5   0.2792778 0.02833460 34.37 0.2217179 0.3368376
## 
## Degrees-of-freedom method: kenward-roger 
## Confidence level used: 0.95 
## 
## $contrasts
## task = reference:
##  contrast            estimate         SE     df t.ratio p.value
##  SALINE1 - ATX0,5 0.106135417 0.05785139 134.00   1.835  0.0688
## 
## task = interference:
##  contrast            estimate         SE     df t.ratio p.value
##  SALINE1 - ATX0,5 0.008027778 0.05701890 133.96   0.141  0.8882
## 
## task = frequency:
##  contrast            estimate         SE     df t.ratio p.value
##  SALINE1 - ATX0,5 0.116305556 0.05694138 133.96   2.043  0.0431
```

## li

```
## Type III Analysis of Variance Table with Satterthwaite's method
##                Sum Sq Mean Sq NumDF  DenDF F value    Pr(>F)    
## pharmaco      1.04536 1.04536     1 165.63 27.3736 5.015e-07 ***
## task          1.42235 0.71117     2 165.32 18.6227 5.082e-08 ***
## pharmaco:task 0.26785 0.13392     2 165.31  3.5069   0.03224 *  
## ---
## Signif. codes:  0 '***' 0.001 '**' 0.01 '*' 0.05 '.' 0.1 ' ' 1
```

```
## $emmeans
## task = reference:
##  pharmaco    emmean         SE    df  lower.CL  upper.CL
##  SALINE1  0.5857500 0.04518307 81.56 0.4958592 0.6756408
##  ATX0,5   0.3042500 0.03296663 31.97 0.2370963 0.3714037
## 
## task = interference:
##  pharmaco    emmean         SE    df  lower.CL  upper.CL
##  SALINE1  0.3239865 0.04725811 57.95 0.2293873 0.4185856
##  ATX0,5   0.2072370 0.03512191 34.11 0.1358695 0.2786045
## 
## task = frequency:
##  pharmaco    emmean         SE    df  lower.CL  upper.CL
##  SALINE1  0.2734926 0.04622099 68.78 0.1812790 0.3657061
##  ATX0,5   0.1764870 0.03512191 34.11 0.1051195 0.2478545
## 
## Degrees-of-freedom method: kenward-roger 
## Confidence level used: 0.95 
## 
## $contrasts
## task = reference:
##  contrast           estimate         SE     df t.ratio p.value
##  SALINE1 - ATX0,5 0.28150000 0.05351761 162.09   5.260  <.0001
## 
## task = interference:
##  contrast           estimate         SE     df t.ratio p.value
##  SALINE1 - ATX0,5 0.11674947 0.05565944 165.85   2.098  0.0375
## 
## task = frequency:
##  contrast           estimate         SE     df t.ratio p.value
##  SALINE1 - ATX0,5 0.09700558 0.05572386 165.81   1.741  0.0836
```

## ca

```
## Type III Analysis of Variance Table with Satterthwaite's method
##          Sum Sq Mean Sq NumDF DenDF F value    Pr(>F)    
## pharmaco 10.991  10.991     1   239  16.522 6.529e-05 ***
## ---
## Signif. codes:  0 '***' 0.001 '**' 0.01 '*' 0.05 '.' 0.1 ' ' 1
```

```
## $emmeans
##  pharmaco   emmean        SE   df  lower.CL upper.CL
##  SALINE1  1.992956 0.2803748 7.31 1.3357112 2.650201
##  ATX0,5   1.424826 0.2928102 8.57 0.7572852 2.092366
## 
## Degrees-of-freedom method: kenward-roger 
## Confidence level used: 0.95 
## 
## $contrasts
##  contrast          estimate       SE  df t.ratio p.value
##  SALINE1 - ATX0,5 0.5681304 0.140946 239   4.031  0.0001
```

## ce

```
## Type III Analysis of Variance Table with Satterthwaite's method
##           Sum Sq Mean Sq NumDF  DenDF F value Pr(>F)
## pharmaco 0.57395 0.57395     1 307.02  2.7102 0.1007
```

```
## $emmeans
##  pharmaco    emmean         SE    df  lower.CL upper.CL
##  SALINE1  0.9685922 0.06562008  7.62 0.8159629 1.121221
##  ATX0,5   1.0640493 0.07804697 12.61 0.8949113 1.233187
## 
## Degrees-of-freedom method: kenward-roger 
## Confidence level used: 0.95 
## 
## $contrasts
##  contrast            estimate         SE    df t.ratio p.value
##  SALINE1 - ATX0,5 -0.09545713 0.05873757 306.6  -1.625  0.1052
```

### << MOVEMENT >>

### —- RT ——

## ci

```
## Type III Analysis of Variance Table with Satterthwaite's method
##               Sum Sq Mean Sq NumDF  DenDF F value    Pr(>F)    
## pharmaco       50359   50359     1 131.38 29.5038 2.613e-07 ***
## task           78823   39412     2 131.38 23.0900 2.551e-09 ***
## pharmaco:task  15101    7550     2 131.30  4.4235   0.01383 *  
## ---
## Signif. codes:  0 '***' 0.001 '**' 0.01 '*' 0.05 '.' 0.1 ' ' 1
```

```
## $emmeans
## task = reference:
##  pharmaco   emmean       SE   df lower.CL upper.CL
##  SALINE1  410.9919 18.47933 8.04 368.4191 453.5648
##  ATX0,5   436.0156 15.72909 4.29 393.4704 478.5609
## 
## task = interference:
##  pharmaco   emmean       SE   df lower.CL upper.CL
##  SALINE1  377.0070 18.45563 8.02 334.4647 419.5493
##  ATX0,5   406.3515 15.55342 4.10 363.5669 449.1360
## 
## task = frequency:
##  pharmaco   emmean       SE   df lower.CL upper.CL
##  SALINE1  420.4503 18.47933 8.04 377.8774 463.0231
##  ATX0,5   497.9421 15.55494 4.10 455.1580 540.7262
## 
## Degrees-of-freedom method: kenward-roger 
## Confidence level used: 0.95 
## 
## $contrasts
## task = reference:
##  contrast          estimate       SE     df t.ratio p.value
##  SALINE1 - ATX0,5 -25.02369 14.16901 131.46  -1.766  0.0797
## 
## task = interference:
##  contrast          estimate       SE     df t.ratio p.value
##  SALINE1 - ATX0,5 -29.34449 13.91429 131.32  -2.109  0.0368
## 
## task = frequency:
##  contrast          estimate       SE     df t.ratio p.value
##  SALINE1 - ATX0,5 -77.49185 13.93145 131.40  -5.562  <.0001
```

## li

```
## Type III Analysis of Variance Table with Satterthwaite's method
##               Sum Sq Mean Sq NumDF  DenDF F value    Pr(>F)    
## pharmaco       34985   34985     1 165.16  7.0672  0.008623 ** 
## task          963867  481934     2 163.98 97.3538 < 2.2e-16 ***
## pharmaco:task  13369    6684     2 163.45  1.3503  0.262035    
## ---
## Signif. codes:  0 '***' 0.001 '**' 0.01 '*' 0.05 '.' 0.1 ' ' 1
```

```
## $emmeans
## task = reference:
##  pharmaco   emmean       SE    df lower.CL upper.CL
##  SALINE1  473.4250 18.36120 28.72 435.8565 510.9935
##  ATX0,5   481.3250 14.60737 12.24 449.5661 513.0839
## 
## task = interference:
##  pharmaco   emmean       SE    df lower.CL upper.CL
##  SALINE1  430.2337 19.05388 28.71 391.2468 469.2206
##  ATX0,5   460.3461 15.24495 13.96 427.6395 493.0527
## 
## task = frequency:
##  pharmaco   emmean       SE    df lower.CL upper.CL
##  SALINE1  600.1343 18.69911 28.75 561.8757 638.3930
##  ATX0,5   653.6239 15.24495 13.96 620.9173 686.3305
## 
## Degrees-of-freedom method: kenward-roger 
## Confidence level used: 0.95 
## 
## $contrasts
## task = reference:
##  contrast          estimate       SE     df t.ratio p.value
##  SALINE1 - ATX0,5  -7.90000 19.26849 162.01  -0.410  0.6824
## 
## task = interference:
##  contrast          estimate       SE     df t.ratio p.value
##  SALINE1 - ATX0,5 -30.11241 20.07495 165.04  -1.500  0.1355
## 
## task = frequency:
##  contrast          estimate       SE     df t.ratio p.value
##  SALINE1 - ATX0,5 -53.48959 20.08058 165.01  -2.664  0.0085
```

## ca

```
## Type III Analysis of Variance Table with Satterthwaite's method
##          Sum Sq Mean Sq NumDF DenDF F value Pr(>F)
## pharmaco 2035.1  2035.1     1 48.64  0.4366 0.5119
```

```
## $emmeans
##  pharmaco   emmean       SE    df lower.CL upper.CL
##  SALINE1  260.0045 7.433911 11.64 243.7521 276.2568
##  ATX0,5   266.6691 9.505588 10.24 245.5576 287.7806
## 
## Degrees-of-freedom method: kenward-roger 
## Confidence level used: 0.95 
## 
## $contrasts
##  contrast          estimate       SE    df t.ratio p.value
##  SALINE1 - ATX0,5 -6.664631 10.79055 60.43  -0.618  0.5391
```

## ce

```
## Type III Analysis of Variance Table with Satterthwaite's method
##          Sum Sq Mean Sq NumDF  DenDF F value  Pr(>F)  
## pharmaco 7287.8  7287.8     1 311.65  5.0133 0.02586 *
## ---
## Signif. codes:  0 '***' 0.001 '**' 0.01 '*' 0.05 '.' 0.1 ' ' 1
```

```
## $emmeans
##  pharmaco   emmean       SE    df lower.CL upper.CL
##  SALINE1  251.3401 6.543839  7.40 236.0327 266.6475
##  ATX0,5   262.2374 7.458805 11.34 245.8808 278.5941
## 
## Degrees-of-freedom method: kenward-roger 
## Confidence level used: 0.95 
## 
## $contrasts
##  contrast          estimate       SE     df t.ratio p.value
##  SALINE1 - ATX0,5 -10.89733 4.911246 311.66  -2.219  0.0272
```

### —- sd RT ——

## ci

```
## Type III Analysis of Variance Table with Satterthwaite's method
##               Sum Sq Mean Sq NumDF  DenDF F value    Pr(>F)    
## pharmaco       36065   36065     1 132.88 15.4676 0.0001345 ***
## task            6587    3293     2 132.64  1.4125 0.2471834    
## pharmaco:task  86186   43093     2 132.53 18.4818 8.324e-08 ***
## ---
## Signif. codes:  0 '***' 0.001 '**' 0.01 '*' 0.05 '.' 0.1 ' ' 1
```

```
## $emmeans
## task = reference:
##  pharmaco    emmean        SE    df  lower.CL  upper.CL
##  SALINE1  177.95815 15.371860 44.20 146.98213 208.93417
##  ATX0,5    62.33175 10.364152 12.50  39.84962  84.81388
## 
## task = interference:
##  pharmaco    emmean        SE    df  lower.CL  upper.CL
##  SALINE1   95.94935 15.339603 44.68  65.04778 126.85091
##  ATX0,5   116.36639  9.998929 10.77  94.30091 138.43188
## 
## task = frequency:
##  pharmaco    emmean        SE    df  lower.CL  upper.CL
##  SALINE1  109.58206 15.371860 44.20  78.60605 140.55808
##  ATX0,5    93.62525 10.001803 10.76  71.55210 115.69839
## 
## Degrees-of-freedom method: kenward-roger 
## Confidence level used: 0.95 
## 
## $contrasts
## task = reference:
##  contrast          estimate       SE     df t.ratio p.value
##  SALINE1 - ATX0,5 115.62640 16.57125 132.91   6.978  <.0001
## 
## task = interference:
##  contrast          estimate       SE     df t.ratio p.value
##  SALINE1 - ATX0,5 -20.41705 16.27729 132.53  -1.254  0.2119
## 
## task = frequency:
##  contrast          estimate       SE     df t.ratio p.value
##  SALINE1 - ATX0,5  15.95682 16.29378 132.72   0.979  0.3292
```

## li

```
## Type III Analysis of Variance Table with Satterthwaite's method
##               Sum Sq Mean Sq NumDF  DenDF F value   Pr(>F)   
## pharmaco       18171 18170.6     1 165.89 10.8464 0.001210 **
## task           16677  8338.4     2 164.53  4.9774 0.007966 **
## pharmaco:task  11044  5522.2     2 163.89  3.2963 0.039489 * 
## ---
## Signif. codes:  0 '***' 0.001 '**' 0.01 '*' 0.05 '.' 0.1 ' ' 1
```

```
## $emmeans
## task = reference:
##  pharmaco   emmean        SE    df  lower.CL upper.CL
##  SALINE1  165.6805  9.979140 47.93 145.61534 185.7457
##  ATX0,5   121.3535  7.596150 18.52 105.42645 137.2806
## 
## task = interference:
##  pharmaco   emmean        SE    df  lower.CL upper.CL
##  SALINE1  126.7982 10.405648 42.52 105.80634 147.7901
##  ATX0,5   109.0755  8.008882 21.02  92.42117 125.7298
## 
## task = frequency:
##  pharmaco   emmean        SE    df  lower.CL upper.CL
##  SALINE1  130.8418 10.188542 45.20 110.32356 151.3600
##  ATX0,5   127.1896  8.008882 21.02 110.53531 143.8440
## 
## Degrees-of-freedom method: kenward-roger 
## Confidence level used: 0.95 
## 
## $contrasts
## task = reference:
##  contrast          estimate       SE     df t.ratio p.value
##  SALINE1 - ATX0,5 44.326975 11.20916 162.04   3.955  0.0001
## 
## task = interference:
##  contrast          estimate       SE     df t.ratio p.value
##  SALINE1 - ATX0,5 17.722717 11.67322 165.80   1.518  0.1309
## 
## task = frequency:
##  contrast          estimate       SE     df t.ratio p.value
##  SALINE1 - ATX0,5  3.652148 11.67837 165.79   0.313  0.7549
```

## ca

```
## Type III Analysis of Variance Table with Satterthwaite's method
##          Sum Sq Mean Sq NumDF  DenDF F value   Pr(>F)   
## pharmaco  17452   17452     1 205.09  11.113 0.001017 **
## ---
## Signif. codes:  0 '***' 0.001 '**' 0.01 '*' 0.05 '.' 0.1 ' ' 1
```

```
## $emmeans
##  pharmaco   emmean       SE    df  lower.CL upper.CL
##  SALINE1  132.7956 8.749607  7.84 112.54592 153.0453
##  ATX0,5   109.5438 9.752503 10.89  88.05193 131.0357
## 
## Degrees-of-freedom method: kenward-roger 
## Confidence level used: 0.95 
## 
## $contrasts
##  contrast         estimate       SE     df t.ratio p.value
##  SALINE1 - ATX0,5 23.25178 7.129553 206.16   3.261  0.0013
```

## ce

```
## Type III Analysis of Variance Table with Satterthwaite's method
##          Sum Sq Mean Sq NumDF  DenDF F value  Pr(>F)  
## pharmaco   3052    3052     1 311.55  5.1953 0.02332 *
## ---
## Signif. codes:  0 '***' 0.001 '**' 0.01 '*' 0.05 '.' 0.1 ' ' 1
```

```
## $emmeans
##  pharmaco   emmean       SE    df lower.CL upper.CL
##  SALINE1  64.06790 4.461590  7.34 53.61712 74.51868
##  ATX0,5   71.14364 5.010013 10.84 60.09730 82.18998
## 
## Degrees-of-freedom method: kenward-roger 
## Confidence level used: 0.95 
## 
## $contrasts
##  contrast          estimate       SE     df t.ratio p.value
##  SALINE1 - ATX0,5 -7.075739 3.129227 311.53  -2.261  0.0244
```
